# Fast and accurate structure probability estimation for simultaneous alignment and folding of RNAs with Markov chains

**DOI:** 10.1186/s13015-020-00179-w

**Published:** 2020-11-13

**Authors:** Milad Miladi, Martin Raden, Sebastian Will, Rolf Backofen

**Affiliations:** 1 Bioinformatics Group, Department of Computer Science, University of Freiburg, Georges-Köhler-Allee 106, Freiburg, Germany; 2 Theoretical Biochemistry Group (TBI), Institute for Theoretical Chemistry, University of Vienna, Währingerstrasse 17, Vienna, Austria; 3 Bioinformatics group (AMIBIO), Laboratoire d’Informatique de l’École Polytechnique (LIX), Institut Polytechnique de Paris (IPP), Batiment Turing, 1 rue d’Estienne d’Orve, Palaiseau, France; 4 Signalling Research Centres BIOSS and CIBSS, University of Freiburg, Schänzlestr. 18, Freiburg, Germany

**Keywords:** RNA secondary structure, Alignment and folding of RNAs, Structural bioinformatics

## Abstract

**Motivation:**

Simultaneous alignment and folding (SA&F) of RNAs is the indispensable gold standard for inferring the structure of non-coding RNAs and their general analysis. The original algorithm, proposed by Sankoff, solves the theoretical problem exactly with a complexity of $O(n^6)$ in the full energy model. Over the last two decades, several variants and improvements of the Sankoff algorithm have been proposed to reduce its extreme complexity by proposing simplified energy models or imposing restrictions on the predicted alignments.

**Results:**

Here, we introduce a novel variant of Sankoff’s algorithm that reconciles the simplifications of PMcomp, namely moving from the full energy model to a simpler base pair-based model, with the accuracy of the loop-based full energy model. Instead of estimating pseudo-energies from unconditional base pair probabilities, our model calculates energies from conditional base pair probabilities that allow to accurately capture structure probabilities, which obey a conditional dependency. This model gives rise to the fast and highly accurate novel algorithm Pankov (Probabilistic Sankoff-like simultaneous alignment and folding of RNAs inspired by Markov chains).

**Conclusions:**

Pankov benefits from the speed-up of excluding unreliable base-pairing without compromising the loop-based free energy model of the Sankoff’s algorithm. We show that Pankov outperforms its predecessors LocARNA and SPARSE in folding quality and is faster than LocARNA.

## Background

In all forms of life, RNAs play essential roles that go beyond coding as messenger RNAs for the synthesis of proteins. Non-coding RNAs (ncRNAs) directly regulate cellular mechanisms, where some are known to be conserved for billions of years [1]. ncRNAs often have only weak sequence conservation, since their (conserved) structure crucially determines their function. Therefore, inferring the conserved secondary structure of homologs—most often, based on RNA alignments, is central for the discovery and annotation of functional RNAs.

RNA structural alignment algorithms can be classified depending on whether they fold and align simultaneously or in turn [2]. The gold standard for computing reliable alignments (and common structures) of RNAs is still the simultaneous algorithm proposed by Sankoff in 1985 [3]. By simultaneously aligning and folding the RNAs, it resolves the vicious cycle that reliable RNA alignments must consider RNA structures (especially for RNAs of medium to low sequence identity [4]), while computational structure prediction is typically unreliable without comparative information. For a pair of RNA sequences, the algorithm finds the optimal alignment and two compatible secondary structures that minimize the total of sequence-alignment distance score and the free energies of the predicted structures. With a run-time complexity of $O(n^6)$ and $O(n^4)$ for memory, the method requires extreme computational resources, such that its application is largely restricted to small instances and data sets. Efficient alignment algorithms are needed for the multiple alignments of the clusters that can be obtained from large scale clustering of data from high-throughput sequencing experiments [5].

Thus, several approaches have adapted Sankoff’s original algorithm to reduce its computational costs. Two main lines of the variants can be distinguished. Methods like Dynalign [6] and FoldAlign [7] reduce the computational complexity by heuristic restrictions, e.g. introducing banding strategies or limiting the maximum size of the comparable sub-structures. Since such methods need to perform expensive energy computations in the nearest neighbor model [8], their applications are still limited without considering heuristic restrictions that in turn could compromise their accuracy.

A highly viable alternative was proposed with the PMcomp algorithm [9], which replaces the nearest neighbor energy model with a still accurate probabilistic model. This model allows to drastically simplify the algorithms, which strongly reduces the computational overhead and supports further algorithmic optimizations in PMcomp’s successors. For reducing the overhead, PMcomp-type algorithms evaluate structures based on base pair probabilities, which are precomputed by McCaskill’s algorithm [10] instead of calculating nearest neighbor energy terms. Moreover, PMcomp-type algorithms such as LocARNA and similar approaches with a probabilistic energy model [11–14] further speed up by reducing the search space. To this end, LocARNA considers only base-pairs that pass a defined probability threshold. This sparsification improves over PMcomp’s complexity of $O(n^6)$ time and $O(n^4)$ space, each, by a quadratic factor (resulting in the $O(n^4)$ time and $O(n^2)$ space requirements of LocARNA).

With the algorithm SPARSE [15], we introduced a second level of sparsification using loop-closing aware recursions to filter based on the joint probability of base-pairs and associated loop-closing base-pairs. This sparsification reduces the time complexity of the alignment algorithm to $O(n^2)$, starting from the precomputed probabilities. The joint probabilities were computed based on our extension of the McCaskill’s algorithm [16].

We emphasize that all these methods, starting with the original Sankoff algorithm, consider only non-crossing structures, even if crossing base pairs occur relatively frequently in physical structures [17]. This is generally justified, since most secondary structures are dominated by non-crossing base pairs; in turn, the limitation to non-crossing structures allows dynamic programming techniques, which are far more efficient and flexible than comparable techniques that consider (even limited forms of) base pair crossings.

In this work, we utilize the joint probabilities in a novel way—not only for strong sparsification as in SPARSE but as well to evaluate RNA structure more accurately in a PMcomp-type algorithm. We start with showing that joint (or equivalently, conditional) probabilities allow to precisely capture structure probabilities in the nearest neighbor energy model. This corresponds to the exact capture of the nearest neighbor energies themselves. Remarkably, while previous work discussed only the stacking base-pair helices [18], we cope with all loop types. Based on the exact model, we suggest careful simplifications, that allow incorporation of the model into alignment and folding (in the variant of the SPARSE algorithm). Based on the novel precise probabilistic model, we propose the novel algorithm Pankov with $O(n^2)$ time complexity. As fundamental novelty over its predecessors, it applies an accurate full-loop energy model for evaluating the structures.

Performing an established benchmark, we show that Pankov is in practice faster than LocARNA *and* significantly improves structure prediction over both SPARSE and LocARNA. Compared to SPARSE, it even improves the sequence alignment quality.

## Methods

### Preliminaries

#### Basic notations

An *RNA sequence* *A* is a string over the alphabet $\{\texttt {A},\texttt {C},\texttt {G},\texttt {U}\}$. A *base pair* *a* *of* *A* is a pair $(a^{\text {L}},a^{\text {R}})$ $(1\le a^{\text {L}} < a^{\text {R}}\le |A|)$ such that the respective sequence positions are complementary, i.e. AU, GC or GU. A *non-crossing RNA structure* $S$ *of* *A*, in the following called *structure*, is a set of base pairs, where each two different base pairs (*i*, *j*) and $(i',j')$ of $S$ do not cross, i.e. $i<i'<j<j'$, and do not share any end, i.e. $i,j,i',$ and $j'$ are pairwise different. To treat the external bases pairs of an RNA structure, we introduce a pseudo-base-pair $a_{0}:=(0,|A|+1)$, which formally encloses all base pairs of *A*.

#### Tree structure

**Fig. 1:**
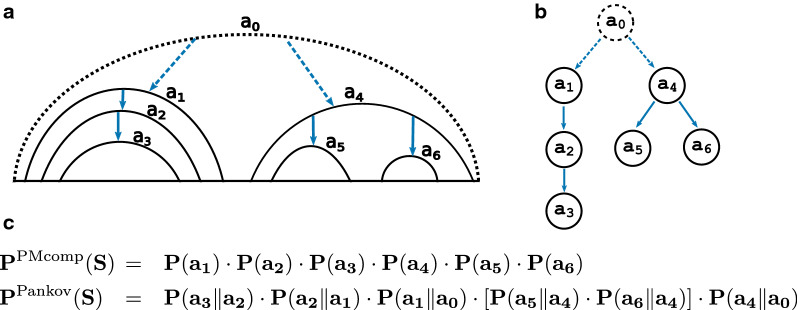
**a**, **b** Illustrations of an exemplary structure *S* and the respective structure tree. PMcomp energy model is composed of the probabilities of base-pairs, shown with plain black arcs and nodes. Pankov energy model additionally incorporates in-loop probabilities of paired base-pairs that are illustrated with blue arrows. **c** Top: PMcomp structure model assume independence between the base-pair probabilities by multiplying the probabilities. Bottom: Pankov uses a loop-aware scheme to compute the probability of the structure that is efficiently calculated from pre-computed loop-conditional probabilities $P(a \Vert a')$ based on the parent-child relation of the base-pairs

A nested RNA secondary structure *S* can be represented as a rooted structure tree, exemplified in Fig. 1a, b, where base-pairs are encoded as nodes and the enclosing base-pairs are the *parents* of the directly enclosed base-pairs. The ${\text {chi}}(a\in S)$ function provides the set of children base pairs that are directly enclosed by a given base pair *a*. Thus, the cardinality of ${\text {chi}}(a)$ is zero for hairpin loops ($a_3, a_5, a_6$ in Fig. 1), at least two for multi-loops ($a_4$) and one otherwise ($a_1, a_2$), which represents stackings, bulges or interior loops. Furthermore, the pseudo base pair $a_{0}$ recursively encloses all base pairs of any structure that can be formed by *A*.

#### Energy and probability of an RNA structure

The *energy of a structure* *S* can be estimated using the nearest neighbor energy model, which is based on a loop-decomposition of the structure, where a loop is defined as the substructure defined by a base pair *a* and its enclosed base pairs ${\text {chi}}(a)$. The energy model provides *contributions* $E^{loop}(a,{\text {chi}}(a))$ *for such loops*, which are summed up, i.e.

1 $$\begin{aligned} E(S)&= \sum _{a \in S}{E^{loop}(a,{\text {chi}}(a))}. \end{aligned}$$

By assuming a Boltzmann distribution of the structures based on the principles of statistical mechanics, there is a bijection between energies and probabilities of structures. Thus, the *probability* *P*(*S*) *of the structure* *S* is related to its energy *E*(*S*) by

2 $$\begin{aligned} P(S)&= \exp {(-E(S)/RT)}/Z \nonumber \\&= \frac{1}{Z} \prod _a Z^{loop}(a,{\text {chi}}(a)), \text { with} \end{aligned}$$

3 $$\begin{aligned} Z^{loop}(a,{\text {chi}}(a))&= \exp (-E^{loop}(a,{\text {chi}}(a))/RT ), \end{aligned}$$

based on its loops’ *Boltzmann weights* (Eq. 3) and the *partition function* $Z = \sum _{S}\exp (-E(S)/RT)$, which is efficiently computed by McCaskill’s algorithm [10]. If inverted, this allows transforming structure probabilities back to energies:

4 $$\begin{aligned} E(S)&= -RT\cdot \log (P(S)) + E^\text {ens}, \end{aligned}$$

where $E^\text {ens} = -RT\cdot \log (Z)$ denotes the ensemble energy. This notation can be further used to derive the common definition of the *probability of a base pair* *a* *P*(*a*) as

5 $$\begin{aligned} P(a)&= \sum _{S\ni a} P(S), \end{aligned}$$

which can be efficiently computed by McCaskill’s algorithm.

### PMcomp assumes independence of base-pair probabilities

#### PMcomp alignment and folding score

The *alignment and folding score* of PMcomp [9], which is assigned to an alignment ${{\mathcal {A}}}$ and RNA structures $S_A$ and $S_B$, can be formulated as

$$\begin{aligned}{}\begin{array}[b]{l} {\text {alignment-score}}^{\text {PMcomp}}(S_A,S_B,{{\mathcal {A}}}) \\ \quad \quad = \sum _{a\in S_A} \Psi ^{A}_{a} + \sum _{b\in S_B} \Psi ^{B}_{b} \\ \quad \quad \quad + \sum _{(i_A,i_B)\in {{\mathcal {A}}}} \sigma (i_A,i_B) + N_{\text {indel}}\gamma . \end{array} \end{aligned}$$

The first two terms define the structural component of the score that is discussed in the next section. The last two terms define the sequence component of the score. $\sigma$ is the base similarity for two matched sequence positions $i_A$ and $i_B$ from sequence *A* and *B*, resp., and $\gamma$ is the gap penalty. $N_{\text {indel}}$ is the number of insertions and deletions in ${{\mathcal {A}}}$.

#### PMcomp structure scoring model

Here, we focus on the structure component of PMcomp’s alignment and folding score, since we want to investigate how well the probabilistic model reflects the thermodynamic energy model. The score of a structure *S* of sequence *A* normalizes and sums up the log-scores of its base pair probabilities, i.e.

6 $$\begin{aligned} \text {score}^{\text {PMcomp}}(S)&= \sum _{a\in S} \Psi ^{A}_{a}\nonumber \\&=\sum _{a\in S}\log \left( P(a)/P_{\min }\right) \nonumber \\&=\log (\prod _{a\in S}P(a))-|S\setminus \{a_{0}\}|\cdot \log (P_{\min }). \end{aligned}$$

Here, base pair probabilities *P*(*a*) are normalized via $P_{\text {min}}$, the minimal probability of a significant base-pair, such that less probable base-pairs are unfavored. The logarithm transfers the probabilistic model back to the energy space similar to Eq. 4.

Putting the normalization term aside, the PMcomp’s structure score contains a notion of structure probability (see first log term on the right of Eq. 6), which we denote with

7 $$\begin{aligned} \text {P}^\text {PMcomp}(S) = \prod _{a\in S} P(a). \end{aligned}$$

Noticeably, base pairing events are assumed to be independent, which is in violation with the underlying Nearest-Neighbor energy model, as we will show next. Thus, PMcomp’s structure score does not relate well with the energy of the respective structure.

### Exact computation of structure probabilities based on conditional loop probabilities

Here we prove that the equilibrium probability of a structure within the ensemble of possible structures can be expressed exactly based on conditional loop probabilities. This provides the theoretical foundation for discussing the Pankov energy model in the subsequent section.

#### **Theorem 1**

*Let* *P*(*S*) *be the probability of structure* *S* *and have* $P(loop(a,{\text {chi}}(a))\mid a)$ *as the conditional probability of the loop in* *S* *closed by base-pair* *a*, *the following equation holds:*

8 $$\begin{aligned} P(S) = \prod _a P( loop(a,{\text {chi}}(a)) \mid a) \end{aligned}$$

#### *Proof*

The free energy of the secondary structure *S* is composed of its loop energies $E^{loop}$ in the nearest-neighbor thermodynamic model (Eq. 1), which implies that its probability can be computed from the respective Boltzmann weights of loops (Eq. 2). Decomposing the right term of Eq. 8 by the partition function $Z_a$ inside the base-pairs *a*, i.e.

9 $$\begin{aligned} Z_a = \sum _{S_a \text { closed by a}} \exp ( - E(S_a) / RT ), \end{aligned}$$

we get:

10 $$\begin{aligned}&\prod _a P( loop(a,{\text {chi}}(a)) | a)\nonumber \\&= \prod _a \frac{\left( \prod _{a'\in {\text {chi}}(a)} Z_{a'}\right) \cdot Z^{loop}(a,{\text {chi}}(a))}{Z_a}\nonumber \\&= \frac{\prod _a\prod _{a'\in {\text {chi}}(a)} Z_{a'}}{\prod _a{Z_a}}\cdot \prod _a{Z^{loop}(a,{\text {chi}}(a))}\nonumber \\&=_{*} \frac{\prod _{a'\ne {a_{0}}} Z_{a'}}{\prod _a{Z_a}}\cdot \prod _a{Z^{loop}(a,{\text {chi}}(a))}\nonumber \\&=_{**} \frac{1}{Z} \prod _a Z^{loop}(a,{\text {chi}}(a)) =_{(\text {Eq.2})} P(S). \end{aligned}$$

($=_*$): Every arc but $a_{0}$ occurs exactly once as a child in the numerator product.

($$=_{**}$$): $$a_{0}$$ encloses all possible structures, thus $$Z_{a_{0}} = Z$$. $$\square$$

Eventually, this work extends and generalizes the approach for canonical helices (only stackings) from [18] to arbitrary secondary structures.

### Pankov structure scoring model

Here, we want to score structures for simultaneous alignment and folding based on the derived Eq. 8, i.e. based on pre-computed conditional loop probabilities, to better reflect the structures’ energy within the overall alignment score. Due to the exponential number of possible multi-loop branchings, a polynomial pre-computation and storing of respective multi-loop probabilities $P(loop(a,{\text {chi}}(a))$ is not feasible. Thus, we propose an approximation of such terms based on pair-in-loop probabilities introduced next.

#### Approximate loop probabilities using pair-in-loop probabilities

To handle arbitrary multi-loops (closed by *a*) with any number and composition of children base pairs ${\text {chi}}(a)$, we restrict computation and storage to all parent-child pairs $a \times a'\in {\text {chi}}(a)$, i.e. we define the *pair-in-loop probability* $P(a' \Vert a)$, in the following abbreviated as *in-loop probabilities*, as

11 $$\begin{aligned} P(a' \Vert a)&= P( a'\in {\text {chi}}(a) \mid a)\nonumber \\&= \frac{P(a, a'\in {\text {chi}}(a))}{P(a)}\nonumber \\&= \frac{\sum _{\begin{array}{c} S\supset \{a,a'\} \wedge a'\in {\text {chi}}(a) \end{array}} P(S)}{P(a)} , \end{aligned}$$

where we calculate the joint probabilities of the form $P(a, a'\in {\text {chi}}(a))$ by an extension of McCaskill’s algorithm introduced in [16], which can be performed in $O(n^3)$ time. To this end, the pair-in-loop information is stored in additional matrices during the partition function computation without increasing the computational complexity. The probability of a base-pair being external, i.e. being enclosed by the pseudo-arc $a_{0}$, is also computed and stored. Within the following, we discuss how to approximate loop probabilities from in-loop probabilities.

##### Pair-in-loop approximation of non-branching-loop probabilities

When using pair-in-loop probabilities to approximate non-branching loop probabilities, i.e. loops with exactly one child base pair, the latter is overestimated since Eq. 11 does not distinguish the loop context of the pair. Therefore, also multi-loops contribute to in-loop probabilities such that it follows

12 $$\begin{aligned} P(a' \Vert a) \ge P( loop(a,\{a'\}={\text {chi}}(a))\mid a ). \end{aligned}$$

##### Scoring based on least stable multi-loop branch

An alternative approximation would be to assign the least probable branch to the whole multi-loop. This can be intuited in the energy space as that least stable branching of the multi-loop dominants formation of the multi-loop.

13 $$\begin{aligned} P_{\text {ML-min}}(loop(a,{\text {chi}}(a))\mid a) = \min \limits _{a' \in {\text {chi}}(a)}P(a' \Vert a). \end{aligned}$$

Due to the same reasons as for non-branching loops, the following relation holds, i.e.

14 $$\begin{aligned} P_{\text {ML-min}}(loop(a,{\text {chi}}(a))\mid a) \ge P( loop(a,{\text {chi}}(a))\mid a ). \end{aligned}$$

##### Assuming multi-loop-branch independence.

As a straightforward approach we assume an independence between the multi-loop branches that are conditioned to be closed under the same base-pair, i.e.

15 $$\begin{aligned} P_{\text {ML-prod}}(loop(a,{\text {chi}}(a))\mid a) = \prod \limits _{a' \in {\text {chi}}(a)}P(a' \Vert a). \end{aligned}$$

#### Weighted overall structure scores

For scoring the structure in the implementation of the Pankov alignment algorithm, we assign the ultimate scoring ${\text {score}}^\text {{Pankov}{}}$ based on the product of multi-loop contributions following Eq. 15, that we designate as the Pankov’s probability of structure.

16 $$\begin{aligned} P^{\text {{Pankov}{}}}(S)&= \prod _{a\in S}\prod _{a'\in {\text {chi}}(a)}P(a' \Vert a) \end{aligned}$$

Similar to PMcomp, the probabilities are incorporated on the logarithmic scale via the $\Phi (a',a)$ function, which also includes a normalization via the bonus term $\beta$. The latter term subsequently balances structure and sequence contributions within the alignment scoring.

17 $$\begin{aligned} \Phi (a',a)&= \log (P(a' \Vert a)) + \beta , \nonumber \\\ {\text {score}}^\text {{Pankov}{}}(S)&= \sum _{a\in S}\sum _{a'\in {\text {chi}}(a)} \Phi (a',a) \nonumber \\&= log(P^{\text {{Pankov}{}}}(S)) + |S\setminus \{a_{0}\}|\cdot \beta \end{aligned}$$

### Pankov alignment approach

The Pankov algorithm keeps track of closing-loop base-pairs in an efficient manner during dynamic programming computation of the score matrices. The matrices are defined similar to the dynamic programming recursions of the SPARSE algorithm [15], which achieve a quadratic time complexity for the alignment by exploiting the sparsity of the in-loop probabilities. Thus, Pankov uses the following matrices:

- $D(a,b)$ for the scores of matching the two base pairs $a=(a^{\text {L}},a^{\text {R}})$ and $b=(b^{\text {L}},b^{\text {R}})$ and aligning the two enclosed subsequences;
- $M^{{a} {b}}(i,k)$ for storing the maximum score of all possible alignments and foldings of the subsequences ${A}[{a^{\text {L}}+1..i}]$ and ${B}[{b^{\text {L}}+1..k}]$ that are under the loops enclosed by *a* and *b*; and
- $I^{{} {}}_\text {A}$ and $I^{{} {}}_\text {B}$ for supporting variability in the helix size via deletion and insertion of base-pairs under the loops closed by *a* and *b* respectively.

The matrix entries can be calculated recursively:

$$\begin{aligned} D(a,b)&= \max {\left\{ \begin{array}{ll} M^{{a} {b}}(a^{\text {R}}-1,b^{\text {R}}-1) \\ I^{{a} {b}}_\text {A}(a^{\text {R}}-1) \\ I^{{a} {b}}_\text {B}(b^{\text {R}}-1) \end{array}\right. } \\ M^{{a} {b}}(i,k)&= \max {\left\{ \begin{array}{ll} M^{{a} {b}}(i-1,k-1) + \sigma (i,k) \\ M^{{a} {b}}(i,k-1) + \gamma \\ M^{{a} {b}}(i-1,k) + \gamma \\ \max _{\begin{array}{c} P(a_1)\ge \theta \\ P(b_1)\ge \theta \\ a_1^{\text {R}}=i\\ b_1^{\text {R}}=k\\ P(a_1,a)\ge \theta '\\ P(b_1,b)\ge \theta ' \end{array}} \left( \begin{array}{l} M^{{a} {b}}(a_1^{\text {L}}\text {-}1,b_1^{\text {L}}\text {-}1)\\ +D(a_1,b_1)\\ +\sigma (a^{\text {L}},b^{\text {L}})\\ +\sigma (a^{\text {R}},b^{\text {R}})\\ +\Phi ({a},a_1)\\ +\Phi ({b},b_1) \end{array}\right) \end{array}\right. } \\ I^{{a} {b}}_\text {A}(i)&= \max {\left\{ \begin{array}{ll} I^{{a} {b}}_\text {A}(i-1) + \gamma \\ \max _{\begin{array}{c} P(a_1)\ge \theta \\ P(a_1,a)\ge \theta '\\ a_1^{\text {R}}=i \end{array}} \left( \begin{array}{l} (a_1^{\text {L}}\text {-}a^{\text {L}}\text {+}1)\cdot \gamma \\ +D(a_1,b)\\ +\Phi ({a},a_1) \end{array}\right) \end{array}\right. } \\ I^{{a} {b}}_\text {B}(k)&= \max {\left\{ \begin{array}{ll}s I^{{a} {b}}_\text {B}(k-1) + \gamma \\ \max _{\begin{array}{c} P(b_1)\ge \theta \\ P(b_1,b)\ge \theta '\\ b_1^{\text {R}}=k \end{array}} \left( \begin{array}{l} (b_1^{\text {L}}\text {-}b^{\text {L}}\text {+}1)\cdot \gamma \\ +D(a,b_1)\\ +\Phi ({b},b_1) \end{array}\right) \end{array}\right. } \end{aligned}$$

Here $\theta$ is the probability threshold and $\theta '$ is the corresponding threshold for the joint in-loop probabilities. Following PMcomp’s energy model, SPARSE calculates $\Phi ({a},a_1)$ as $\Psi {a_1}$ (independent of the enclosing base pair *a*)—in Pankov we make use of the full flexibility.

The asymptotic time complexity of the Pankov alignment algorithm is $O(n^2)$. Similar to SPARSE, the base pairing and joint in-loop probabilities need to be computed only once for each sequence. This is important for computing multiple alignments, using a pairwise aligner like Pankov in a progressive scheme (e.g. via mlocarna tool from the LocARNA software package). The Pankov implementation of the recursions keeps track of *both* ends of the base-pairs, while in SPARSE the tracking is relaxed and *M* matrices with common left-ends are combined for further speed-up, although the complexity is not affected. So in practice, compared to SPARSE, Pankov’s run-time is slightly increased.

### Alignment with domain insertion and deletion

The recursions of Pankov can be extended to allow for the deletion of an entire branch of one of the two predicted structures, which is then aligned to a gap in the other structure. This is similar to the Dynalign-II support for the insertion/deletion of domains [19]. Below, the additional two cases of Pankov’s *M*-recursion are shown.

$$\begin{aligned} M^{{a} {b}}(i,k)&= \max {\left\{ \begin{array}{ll} \quad \;\; [..] \\ \max _{\begin{array}{c} P(a_1)\ge \theta \\ P({a_1},{a})\ge \theta '\\ a_1^{\text {R}}=i\\ a_1^{\text {R}}-a_1^{\text {L}}<L_D \end{array}} \left( \begin{array}{l} M^{{a} {b}}(a_1^{\text {L}}-1,k)\\ +2\gamma \\ +D(a_1,-)\\ +\Phi ({a},a_1) \end{array}\right) \\ \max _{\begin{array}{c} P(b_1)\ge \theta \\ P({b_1},{b})\ge \theta '\\ b_1^{\text {R}}=k\\ b_1^{\text {R}}-b_1^{\text {L}}<L_D \end{array}} \left( \begin{array}{l} M^{{a} {b}}(i,b_1^{\text {L}}-1)\\ +2\gamma \\ +D(-,b_1)\\ +\Phi ({b},b_1) \end{array}\right) \end{array}\right. } \end{aligned}$$

The cost of deleting/inserting both ends of the enclosing base pairs of the domain, $a_1$ or $b_1$, is $2\gamma$. The extended *D* matrix entries, i.e. $D(a_1,-)$ and $D(-,b_1)$, are initialized with the cost for the deletion/insertion of the enclosed domain. The maximum allowed size of a deleted domain is limited by the parameter $L_D$, since arbitrary large domain indels are unlikely. This keeps the run-time increase moderate and allows to turn off the feature of domain insertion/deletion by $L_D=0$.

## Results and discussion

### Evaluation of the probabilistic energy models

#### The evaluation procedure

We evaluated the agreement between the reference and the probabilistic free energy models. Having the Turner [8] nearest-neighbor full energy model as the reference, we compared the performance of PMcomp’s base-pair independence model and Pankov’s loop-based conditional probability model. The Sankoff-like algorithms maximize (or minimize) the sum of structure and sequence alignment scores over the space of possible formations of alignments and structure. Hence, a higher correlation between the calculated energies of a model with the reference free energy values indicates better modeling of the structure score, that is expected to perform better for the task of RNA simultaneous alignment and folding.

We developed an evaluation procedure to measure the level of agreement between the probabilistic models and the reference energy model with correlation coefficients. The procedure performs these steps for an input RNA sequence: (i) suboptimal secondary structures are generated using RNAsubopt method [20], for the range of the minimum free energy structure up to 5kcal/mol (-e=5 -s) and 500 suboptimals. (ii) for each suboptimal structure, the probability or free energy is calculated according to the described energy models and structure scores (iii) Over the set of suboptimal structures, the Spearman’s rank correlation coefficient between the reference RNAsubopt’s free energies and the free energies/scores of the models are computed.

The five approximation variants of the probabilistic models are evaluated which compute the probability or score of a structure. More precisely, these variants are computed according to the equations $\text {P}^\text {PMcomp}$ (Eq. 7), ${\text {score}}^{\text {PMcomp}}$ (Eq. 6), $P^{\text {{Pankov}{}}}$ using $P_{\text {ML-min}}$ (Eq. 13), $P^{\text {{Pankov}{}}}$ using $P_{\text {ML-prod}}$ (Eq. 15) and ${\text {score}}^\text {{Pankov}{}}$ using $P_{\text {ML-prod}}$ and $\beta$ terms (Eq. 17). The structure probabilities are transferred to the energy dimension according to Eq. 4. For consistency in the representation, the scores were also scaled with a similar scheme. As a monotonic function, the energy scale transformation does not affect the absolute rank correlations in neither of the cases.

The minimal significance probability $P_{\min }$ in Eq. 6 was set to $1/(2\cdot \text {sequence-length})$, this is used for the PMcomp score scheme of LocARNA. The bonus balance term $\beta$ from Eq. 17 was set to 1.5, a bonus of zero makes the rank correlation of ${\text {score}}^\text {{Pankov}{}}$ same as the rank correlations of $P^{\text {{Pankov}{}}}$ using $P_{\text {ML-prod}}$. However, a non-zero value is needed to balance the total alignment score by appropriately shifting the energy. The base-pair and conditional in-loop probabilities were computed using the extended implementation of McCaskill’s algorithm (see methods). The run-time complexity for calculating these values for a structure, using the precomputed matrices, are linear to the number of base-pairs in structure (*O*(|*S*|)) as well as the length of the sequence.

#### Evaluation of real ncRNAs

**Fig. 2:**
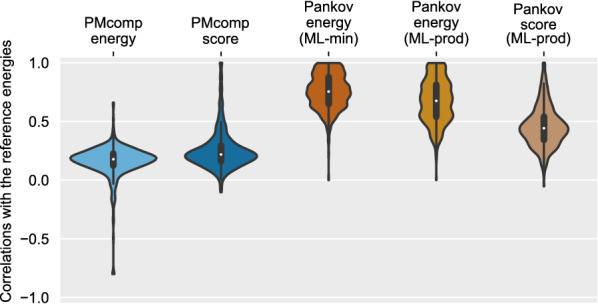
The distribution of rank correlation for evaluating the energy models over around 500 RNA sequences from RNAstrand database. Spearman’s rank correlations are computed for each sequence, between the reference and the models’ approximations of the suboptimal energies

The described evaluation procedure was repeated for the set of RNA sequences obtained from the RNAstrand database (sequence length [30–200] nucleotides, entries without ambiguous or spurious characters). Figure 2 shows the distribution of the rank correlation evaluation of the sequences.

**Fig. 3:**
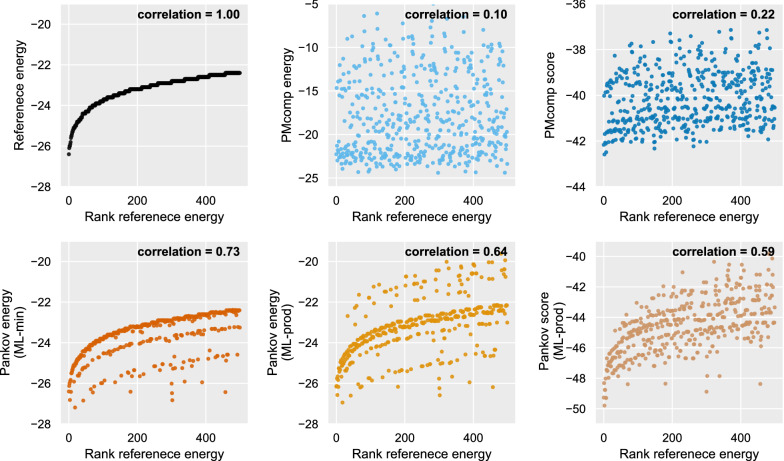
Evaluation of the agreement between the structure probabilistic and scoring models with the reference energies for a tRNA transcript. Each dot corresponds to a suboptimal structure of the tRNA. The structures are sorted according to reference free energy in the x-axis, computed by Vienna package RNAsubopt tool. The probabilities and scores are scaled to the energy dimension (Eq. 4). Annotated correlations of each panel are the Spearman’s correlation coefficients between the x and y axes. Each correlation corresponds to an individual entry within the corresponding distributions that are shown as violin plots in Fig. 2

To inspect the model agreements in details, we visualized the output on a sample tRNA transcript of RNAstrand (ID: SPR_00633) in Fig. 3. An evaluation for the top 500 suboptimal structures of lowest free energies is shown. As can be seen in Fig. 3, the Pankov’s in-loop-probability-based models (bottom row of Fig. 3) perform best in preserving the reference free energy ranks. It is also notable that the energy scaled values are precisely scaling back to the range of reference free energies.

The scatter plot for Pankov energy (ML-min) in Fig. 3 confirms the relation for the probability of multiloops that was presented in the methods section (Eq. 14), $P^{\text {{Pankov}{}}}$ with a ${\text {ML-min}}$ approximation is bounded by the exact probability of the structure such that the approximated energies are always less than or equal to the reference energies.

### Alignment performance evaluation

We evaluated our implementation of Pankov alignment algorithm on the pairwise alignment benchmark set, Bralibase 2.1 [21]. For the evaluated methods, the sparsification probability thresholds were set similar. Namely, LocARNA, SPARSE and Pankov with the minimum base-pair probability $\theta$ to 0.001 (option-p). For SPARSE and Pankov the in-loop probability threshold $\theta '$ was set to 0.0001 (option–prob-basepair-in-loop). The sequence-structure balance term $\beta$ of Pankov’s score (Eq. 17) was set to 1.5, the chosen among the values 1, 1.5, 2 and 2.5 posing a fair balance for the average of sequence and structure scores. The Matthews Correlation Coefficient (MCC) performance was stable for $\beta$’s value of 1.5 and larger values (Additional file 1: Figure S1).

**Fig. 4:**
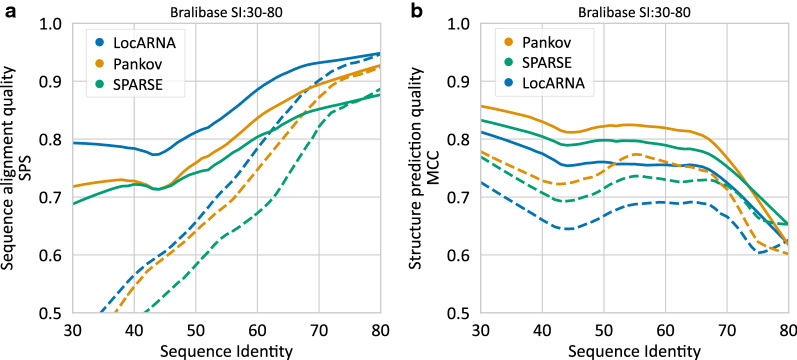
Comparison of the alignment performance on the Bralibase 2.1 pairwise benchmark set K2. **a** The sequence-level quality is measured as sum-of-pairs-score (SPS) by comparing the alignment edges of the reference and predicted alignments. **b** The structure prediction quality is measured as Matthews Correlation Coefficient (MCC) by comparing the base-pairs of the reference structure with the predicted structures. The solid lines depict all families and the dashed lines depict the subset without tRNA and the two rRNA families

Figure 4a, b show the performance comparison in term of sequence alignment quality sum-of-pairs-score (SPS) and structure prediction quality by Matthews Correlation Coefficient (MCC) [22]. To mediate the Bralibase curve “dent” effect [23], the visualization was done for sequence pairs of sequence identity (SI) between 30 to 80% to avoid a curve dent around SI-80 that seems to be mainly caused by enforcing a continuous curve over a quasi-heterogeneous distribution. Entries with a higher SI are not of particular interest, as they mostly perform fine also using the structure-unaware alignment algorithms. Furthermore, the dashed curves in Fig. 4 correspond to the subset of the benchmark by excluding the three ribosomal and tRNAs families. These families are shown to be moderately overrepresented in the Braliabase and could overweight in the overall performance, especially on lower sequence identity range [23].

In the aspect of execution time, Pankov is overall faster than LocARNA and slower than SPARSE since Pankov implements the exact loop-closing track of the alignment recursions (see methods). Our implementation of Pankov had an average run time of 1.8 seconds on Bralibase K2 instances, when running on a AMD Opteron 2.1 GHz processor; this compares to respective average run times of 3.9 and 0.7 seconds of LocARNA and SPARSE. As can be seen in Fig. 4, Pankov considerably improves structure prediction over both SPARSE and LocARNA. Compared to its predecessor SPARSE, it even improves the sequence alignment quality.

#### Family-wise analysis

**Fig. 5:**
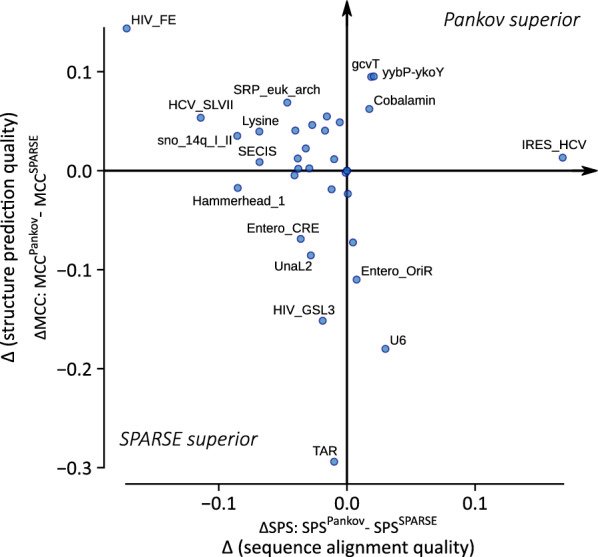
Family-wise performance analysis on the Bralibase 2.1 pairwise benchmark set K2. (X-axis) The average difference between the Pankov and SPARSE for the sequence alignment quality metric SPS is computed per RNA family. (Y-axis) Similar to the X-axis but for the structure prediction metric MCC. Families with extreme differences are labeled. Pankov uses the superior estimation of the energy model while SPARSE applies PMcomp’s model (see Fig. 2). Pankov’s alignments gain in term of structure prediction for the majority of families

We further compared the performance of SPARSE and Pankov in a family-wise manner, to find out which families benefit most from the incorporation of a more accurate energy model. Fig. 5 illustrates the difference ($\Delta$) between quality metrics of SPARSE and Pankov which are averaged per family for Bralibase K2 entries with an SI less than 80%. The majority of families gain in the term of structure prediction from Pankov’s accurate energy model. Furthermore, *IRES_HCV* and the riboswitch families *yybP-ykoY*, *Cobalamin* and *glycine (gcvT)* have an improved sequence alignment quality (SPS) as well. A couple of families performed worse on average in structure prediction metric using the more accurate energy model of Pankov. Overall these families tend to be short and accommodate simple non-branching structures, according to Rfam’s reference structures.

#### The effect of domain indel feature

The possibility of deleting or inserting an entire domain (i.e. a branch of the structure tree) is beyond the original Sankoff’s algorithm. In Sankoff’s model the structures are constrained to have the same branching topology. The Pankov extended version with domain indel support ($L_D=70$) had about 80% increase of the runtime. On the other side, the average alignment quality metrics (both SPS and MCC) are mainly improved or remained unchanged (Additional file 1: Figure S2).

## Conclusion

Sankoff’s algorithm is the reference standard for simultaneous alignment and folding (SA&F) of RNAs. While the theoretical work integrates the full loop-based nearest-neighbor energy model, the derived algorithms mostly implement a simplified or limited structure energy models or restrict on the alignment and structure formation possibilities, to reduce the high computational complexity. PMcomp proposed a probabilistic light-weight energy model. This empowers the PMcomp-like methods to strongly reduce the computational overhead of the exact thermodynamic folding details and allows further algorithmic optimizations and sparsification based on the equilibrium probability of the base-pairs.

Here we showed that PMcomp’s energy model assumes a level of independence between the base-pairing events, which violates the underlying nearest neighbor energy model. To solve this issue, we demonstrated an exact way to compute the probability of an RNA secondary structure from the decomposed loop-probabilities. To circumvent the computational complexity of multi-loop cases, we introduced an energy model to accurately approximate this loop decomposition in an efficient way using the precomputed in-loop probabilities. Our proposed energy model takes care of the nearest-neighbor thermodynamic rule. It was further empirically validated that the novel model has a much closer agreement with the full-loop energy model, based on the dataset of real non-coding RNAs. Using this energy model, we proposed the Pankov algorithm for pairwise simultaneous alignment and folding of RNAs. Benchmark results show that the implementation of Pankov outperforms its predecessors on predicting the secondary structure from the pairs of homologous RNAs.

The concept of conditional and joint in-loop probabilities has some parallels to the production rules of Stochastic Context-Free Grammars (SCFG) that can encode base-pair relations differently [24], they have also been used to solve the SA&F problem [25]. The overhead of treating various nucleotides separately during the alignment procedure is similar to the invocation of the full loop-based energy model, which restrains the implementation towards using simplified grammars that may not benefit from the power of thermodynamic rules. In our proposed model, the probabilistic terms are obtained from the thermodynamic partition function, so the probabilistic transition rules are straightforward and do not need to deal with individual types and combinations of nucleotides separately.

Pankov, to the best of authors’ knowledge, is the first SA&F method that dissociates the loop computation details from the alignment and prediction step to efficiently solve the target problem without substantially compromising the power of underlying thermodynamic rules.

## Supplementary information

**Additional file 1: Fig. S1.** Evaluation of the bonus score effect on the combination of sequence and structure score for the alignment of Bralibase dataset. The Matthews Correlation Coefficient (MCC) performance is stable for β's value of 1.5 and larger values. **Fig. S2.** (A) Family-wise performance analysis of the benchmark set with and without enabling the option domain insertion and deletion. (B) An example of Pankov predicted structures by aligning two TPP (THI element) riboswitches. Correctly alignment columns are colored. The light green bases are predicted by Pankov and SPARSE, while the dark green and cyan regions are only predicted by Pankov. The highlighted stem-loop is deleted as a domain in the Pankov's prediction. The cyan nucleotides have more than 97% sequence conservation according to the Rfam family TPP (RF00059), a.k.a. THI-box riboswitch, and can only be aligned once the domain insertion-deletion option is enabled.

## Data Availability

Pankov is developed as a branch of the LocARNA package and available at https://github.com/BackofenLab/Pankov. The data and analysis of the correlation analysis (Figs. 2 and 3) are provided within the Github repository.

## Abbreviations

MCC: Matthews Correlation Coefficient
SA&F: Simultaneous alignment and folding
SI: Sequence identity
SPS: Sum-of-pairs score
