# Supplementary material for: Fast and accurate structure probability estimation for simultaneous alignment and folding of RNAs with Markov chains
Source: Algorithms Mol Biol. 2020 Nov 13;15:19. doi: 10.1186/s13015-020-00179-w (PMC7666477; doi:10.1186/s13015-020-00179-w)
Supplement: Supplementary file 1 — Additional file 1: Fig. S1. Evaluation of the bonus score effect on the combination of sequence and structure score for the alignment of Bralibase dataset. The Matthews Correlation Coefficient (MCC) performance is stable for β's value of 1.5 and larger values. Fig. S2. (A) Family-wise performance analysis of the benchmark set with and without enabling the option domain insertion and deletion. (B) An example of Pankov predicted structures by aligning two TPP (THI element) riboswitches. Correctly alignment columns are colored. The light green bases are predicted by Pankov and SPARSE, while the dark green and cyan regions are only predicted by Pankov. The highlighted stem-loop is deleted as a domain in the Pankov's prediction. The cyan nucleotides have more than 97% sequence conservation according to the Rfam family TPP (RF00059), a.k.a. THI-box riboswitch, and can only be aligned once the domain insertion-deletion option is enabled. [file 13015_2020_179_MOESM1_ESM.pdf]

Fast and accurate structure probability estimation for simultaneous alignment and folding of RNAs with Markov chains

—

Supplementary document

Milad Miladi, Martin Raden, Sebastian Will, Rolf Backofen

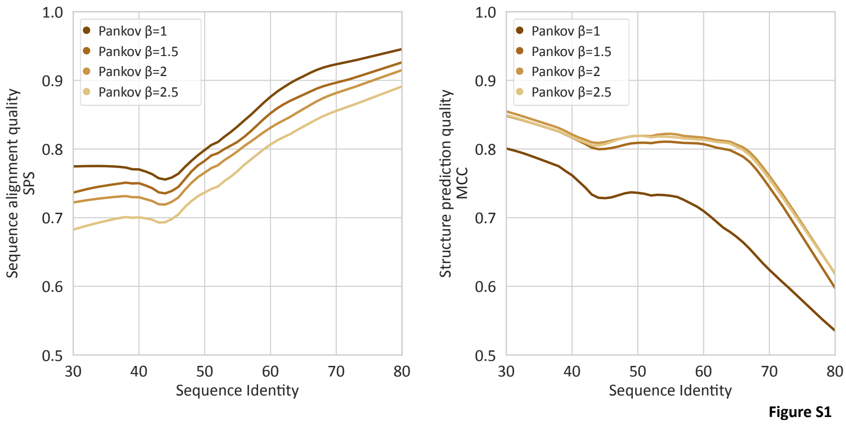

Figure S1: Evaluation of the bonus score effect on the the combination of sequence and structure score for the alignment of Bralibase dataset. The Matthews Correlation Coefficient (MCC) performance is stable for β's value of 1.5 and larger values

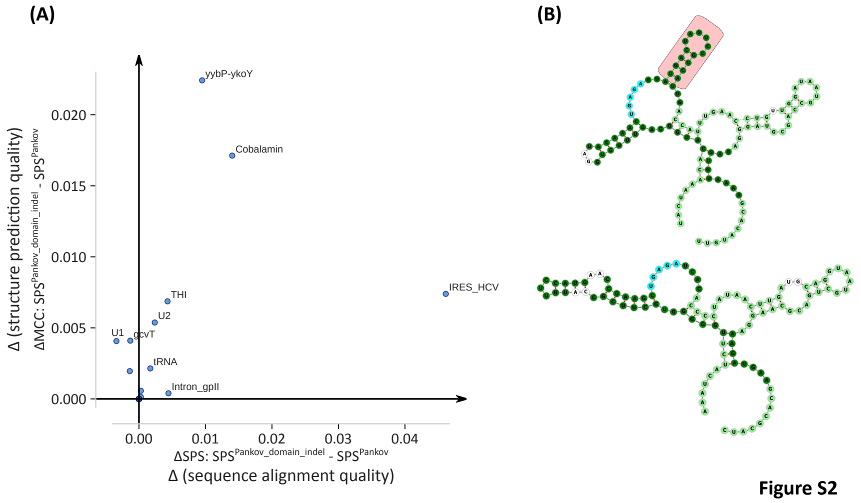

Figure S2

Figure S2: **(A)** Family-wise performance analysis of the benchmark set with and without enabling the option domain insertion and deletion. **(B)** An example of Pankov predicted structures by aligning two TPP (THI element) riboswitches. Correctly alignment columns are colored. The light green bases are predicted by Pankov and SPARSE, while the dark green and cyan regions are only predicted by Pankov. The highlighted stem-loop is deleted as a domain in the Pankov's prediction. The cyan nucleotides have more than 97% sequence conservation according to the Rfam family TPP (RF00059), a.k.a. *THI-box riboswitch*, and can only be aligned once the domain insertion-deletion option is enabled
